# Event and Comment

**Published:** 1912-06-15

**Authors:** 


### DENTAL REGISTER.

**Vol. LXVI. June 15, 1912. No. 6**

### EVENT AND COMMENT.

HOW EDUCATE OUR SPECIALISTS. The medical profession has for a long time tried to devise some way of developing its specialists with some substantial and rational reason for their existence. So far there seems no better way than to give them the regulation medical course with the Medical Doctor degree, and then allow the graduate to specialize in the knowledge and skill necessary to justify him in announcing himself as a specialist. This means that a recent graduate may announce himself as a specialist at once and by confining his work to his specialty he in time is recognized by the profession and the public as a specially qualified physician in certain diseases. This self-educating process is a slow one as it takes time to convince the public that one is a real specialist. The other method is for the graduate to seek hospital or personal clinical advantages where he can observe and study the methods of experts until he can make them his own. If one gives sufficient time and is careful to select the higher sources of training he more quickly assumes and is credited with the arts of the specialist.

In dentistry we are following largely the same method as obtains in medicine. It is true we have post-graduate schools in orthodontia, mechanical and operative technics, which replace the hospitals of the medical practitioners; and we seem satisfied to recognize as a specialist any one who may have attended a six or eight weeks course in one of these schools, and then announces himself as a specialist, and we make no protest, and by common consent or indifference he is a specialist, and we leave it all to him to develop skill and knowledge to justify his assumption. Is it right that we should, as a profession, countenance such haphazard methods? Should we not enact into our statutes some legal qualification which shall be an assurance to the people that any one holding himself out as a specialist has honestly met the requirements which give him the right to so announce himself? People go to the specialist with confidence and pay him larger fees than they ever dream of paying the ordinary practitioner because they feel their case is a peculiarly intricate one to diagnose or has some special difficulties in treatment which the ordinary practitioner can not handle.

Now since any one may announce himself as a specialist and the people may not be wise enough to decide whether he is really a skillful practitioner or only a pretender, should not the specialist have a special license from the State Registration Boards or should he not have at least a certificate or degree from a school of recognized standing? If something of this kind is not done it would seem that there will in time arise a confusion of so-called specialties which will be demoralizing to an extreme so far as standards of excellence are concerned. The new specialty of Prophylaxis seems most likely to be *"still born,"* if the "trained nurse" is turned loose upon the public, as seems inevitable. Oral Prophylaxis can no more be adequately administered by the "trained nurse" than the automobilist can safely conduct a flying machine. The dental profession and its specialties can not accept the prophylaxis specialist unless he has had preliminary and fundamental training, supplemented by specific knowledge and concentrated manipulative skill. Can an education or training of such dimensions be possible unless placed upon an accepted pedagogical basis? The best which we now know is embodied in our dental schools. Again can the dental colleges undertake to train dental nurses with a less comprehensive curriculum than is now exacted for the general practitioner. These questions must be settled in the near future. Shall we act wisely or blunder?

